# Objectively Measured Physical Activity Is Associated With Body Composition and Metabolic Profiles of Pacific and New Zealand European Women With Different Metabolic Disease Risks

**DOI:** 10.3389/fphys.2021.684782

**Published:** 2021-05-26

**Authors:** Joanne Slater, Rozanne Kruger, Jeroen Douwes, Wendy J. O’Brien, Marine Corbin, Jennifer L. Miles-Chan, Bernhard H. Breier

**Affiliations:** ^1^School of Sport, Exercise and Nutrition, Massey University, Auckland, New Zealand; ^2^Research Centre for Hauora and Health, Massey University, Wellington, New Zealand; ^3^Human Nutrition Unit, School of Biological Sciences, University of Auckland, Auckland, New Zealand; ^4^Riddet Centre of Research Excellence, Palmerston North, New Zealand; ^5^Microbiome Otago, University of Otago, Dunedin, New Zealand

**Keywords:** physical activity, metabolic health, obesity, body composition, women’s health, insulin, body fat

## Abstract

**Objective:** To assess associations between physical activity (PA), body composition, and biomarkers of metabolic health in Pacific and New Zealand European (NZE) women who are known to have different metabolic disease risks.

**Methods:** Pacific (*n* = 142) or NZE (*n* = 162) women aged 18–45 years with a self-reported body mass index (BMI) of either 18.5–25.0 kg⋅m^–2^ or ≥30.0 kg⋅m^–2^ were recruited and subsequently stratified as either low (<35%) or high (≥35%) BF%, with approximately half of each group in either category. Seven-day accelerometery was used to assess PA levels. Fasting blood was analysed for biomarkers of metabolic health, and whole body dual-energy X-ray absorptiometry (DXA) was used to estimate body composition.

**Results:** Mean moderate-to-vigorous physical activity (MVPA; min⋅day^–1^) levels differed between BF% (*p* < 0.05) and ethnic (*p* < 0.05) groups: Pacific high- 19.1 (SD 15.2) and low-BF% 26.3 (SD 15.6) and NZE high- 30.5 (SD 19.1) and low-BF% 39.1 (SD 18.4). On average Pacific women in the low-BF% group engaged in significantly less total PA when compared to NZE women in the low-BF% group (133 cpm); no ethnic difference in mean total PA (cpm) between high-BF% groups were observed: Pacific high- 607 (SD 185) and low-BF% 598 (SD 168) and NZE high- 674 (SD 210) and low-BF% 731 (SD 179). Multiple linear regression analysis controlling for age and deprivation showed a significant inverse association between increasing total PA and fasting plasma insulin among Pacific women; every 100 cpm increase in total PA was associated with a 6% lower fasting plasma insulin; no significant association was observed in NZE women. For both Pacific and NZE women, there was an 8% reduction in fasting plasma insulin for every 10-min increase in MVPA (*p* ≤ 0.05).

**Conclusion:** Increases in total PA and MVPA are associated with lower fasting plasma insulin, thus indicating a reduction in metabolic disease risk. Importantly, compared to NZE, the impact of increased total PA on fasting insulin may be greater in Pacific women. Considering Pacific women are a high metabolic disease risk population, these pre-clinical responses to PA may be important in this population; indicating promotion of PA in Pacific women should remain a priority.

## Introduction

Obesity is a global public health problem. The prevalence in New Zealand (NZ) is among the highest in the world (Ministry of Health New Zealand, 2020), with 31% of the population aged ≥ 15 years considered to be obese [body mass index (BMI) ≥ 30.0 kg⋅m^–2^, or International Obesity Task Force equivalent for 15–17 years] (Ministry of Health New Zealand, 2020). Obesity is described as an “excessive fat accumulation that may impair health” (World Health Organisation, 2016a) and the World Health Organization has defined standardised cut-points for overweight (BMI ≥ 25.0 and < 30) and obesity (BMI ≥ 30.0). However for a given BMI, factors such as age, sex and ethnicity influence body fat percentage (BF%) and distribution, thus individuals with the same BMI may have different metabolic disease risks (Kramer et al., 2013; Oliveros et al., 2014). Global and NZ trends show a significantly greater rise in obesity prevalence among women, when compared to men, with major BMI gains between the ages of 20–40 years (The GBD 2015 Obesity Collaborators, 2015). Furthermore, the global obesity prevalence in women nearly tripled from 6% in 1975 to 15% in 2014 (NCD Risk Factor Collaboration, 2016); in 2008, this was projected to rise to 21% by 2025 (Kelly et al., 2008; NCD Risk Factor Collaboration, 2016). Alarmingly, current estimations of obesity among NZ women already surpass this figure, with 32% currently classed as obese (Ministry of Health New Zealand, 2020). Obesity disproportionally effects disadvantaged socioeconomic (Zhang and Wang, 2004) and ethnic groups (Hales et al., 2020). In NZ, adults living in the most deprived areas are 1.6 times more likely to have obesity compared to adults living in the least deprived areas. Obesity prevalence is highest among Māori (the indigenous population of NZ) (48%) and Pacific (70%) women (Ministry of Health New Zealand, 2020), which is of particular concern as obesity is associated with a substantial burden of disease (Wagner and Brath, 2012). The high prevalence of obesity among Māori may, among other reasons (e.g., deprivation), be due to the impact of colonisation, which has resulted in a loss of traditional food gathering practices and places, as well as the introduction of new foods (Gray, 2003; Ministry of Health NZ, 2006). In addition, current individualistic approaches of western medicine to obesity management are not culturally aligned with Indigenous people’s holistic health believes and cultural practices, hampering the effectiveness of public health interventions (Bell et al., 2017). Some of these factors may also play a role in the high prevalence of obesity among Pacific people in NZ.

Obesity reflects a state of positive energy balance and is associated with altered glucose and lipid metabolism that may lead to an increased risk of non-communicable diseases, such as type 2 diabetes (T2D), some cancers and cardiovascular disease (CVD) (Wagner and Brath, 2012). Hence, the long-term health impact of obesity is considerable, especially for women of childbearing age, as increased adiposity is also associated with increased obesity risk for the next generation (Eriksson et al., 2014).

Currently, physical activity (PA), together with diet, are the cornerstones of obesity intervention and prevention. Many of the health benefits associated with PA are mediated through its ability to improve glucose and lipid metabolism (Timmermans et al., 2006). Current PA guidelines include promoting ≥ 30 min of moderate (or greater) intensity activity on at least five days of the week (Ministry of Health New Zealand, 2015; U.S. Department of Health and Human Services, 2018). However, currently less than one third (31%) of the world population are meeting these recommendations (Hallal et al., 2012); this is particularly the case in women with obesity (Tudor-Locke et al., 2010). For example, in NZ, only 48% of women met PA recommendations in 2019/20 [Pacific and New Zealand European (NZE) women 39 and 51%, respectively] (Ministry of Health New Zealand, 2020). In agreement with global trends, NZ women were less physically active when compared to NZ men (55% met PA recommendations) and NZ adults living in the most deprived areas are least likely to meet these PA guidelines (Ministry of Health New Zealand, 2020). Even more concerning, is that 25% of Pacific and 14% of NZE women fail to perform even 30 min of moderate PA per week, classifying them as inactive (Ministry of Health New Zealand, 2020). It is therefore not surprising that physical inactivity is currently classified as the fourth leading risk factor for global mortality (World Health Organisation, 2016b). Recent research suggests that total PA volume accumulated throughout the day may be as important as time spent in moderate-to-vigorous physical activity (MVPA) for lowering markers of metabolic disease risk [e.g., fasting insulin and high density lipoprotein (HDL]) (Ekelund et al., 2007; Swindell et al., 2018). To date, research on PA and its association with metabolic health has focused on healthy subjects (Healy et al., 2008; Nelson et al., 2013; Knaeps et al., 2016) as well as populations with high metabolic disease risk (Ekelund et al., 2016; Swindell et al., 2018). However, most research has been carried out in people of European ancestry who have a lower metabolic disease incidence, including CVD and T2D, compared to many other ethnic groups (Ekelund et al., 2007, 2009; Henson et al., 2013; Hamasaki et al., 2015; Swindell et al., 2018). Considering women of Pacific descent are at increased risk of obesity, T2D and CVD compared with women of European descent (Sundborn et al., 2008; Ministry of Health New Zealand, 2020), and that increasing total PA is associated with metabolic health, then encouraging movement of all intensities, rather than focusing solely on MVPA may be a more achievable public health measure for such populations. However, evidence for this is limited.

The aim of this cross-sectional study was to: (1) determine if objectively measured PA was associated with different metabolic health risk and body fat profiles in a population of healthy, normal-weight and obese women, aged 18–45 years; and (2) determine whether these associations differ between ethnic groups (Pacific and NZE). We hypothesize that PA patterns account for ethnic differences in metabolic health, independent of level of deprivation and body composition.

## Materials and Methods

### Participants and Setting

Healthy Pacific and NZE women in NZ were invited to take part in the cross-sectional study, PRedictors linking Obesity and the gut MIcrobiomE (PROMISE) (Kindleysides et al., 2019). The methodological details of the PROMISE study have been published previously (Ministry of Health New Zealand, 2020).

In brief, inclusion criteria included: women aged 18–45 years, post-menarche and pre-menopausal (as defined by regular menstrual cycles over the last year) in self-reported general good health. Participants were also required to self-identify their ethnicity as follows: being of Pacific ethnicity (self-identified), which required having at least one parent of Pacific ethnicity (no minimum time for living in NZ was required), and NZE ethnicity, which required both parents of European descent (NZ or overseas born) and having themselves lived in NZ for ≥ 5 years. Participants’ self-reported height and weight was collected to enable BMI calculation and categorisation. Women who had a BMI within the predefined normal or obese BMI ranges (BMI ≥ 18.5 to < 25.0 kg⋅m^–2^ and ≥ 30.0 kg⋅m^–2^, respectively) were invited to participate with the aim of recruiting approximately equal numbers in each BMI/ethnic group. In recognition that people with the same BMI can have substantial heterogeneity of body fat and metabolic disease risk factors (Dickey et al., 1998; Kramer et al., 2013; Oliveros et al., 2014), participants were subsequently classified into two groups based on BF%: low-BF% (<35%) and high-BF% (≥35%). BF% cut-offs were derived from the American Association of Clinical Endocrinologists and American College of Endocrinology guidelines (obesity in women > 35%) (Dickey et al., 1998; Oliveros et al., 2014; Jo and Mainous, 2018). Exclusion criteria were pregnancy or lactation and presence of any diagnosed chronic illness (e.g., T2D and CVD).

Eligible participants attended our research clinic on two occasions, 11–14 days apart. All participants provided written informed consent prior to participation. The study was approved by the Health and Disability Ethics Committee (HDEC, reference: 16/STH/32) and the trial was registered at anzctr.org.au (ACTRN12618000432213).

### Measurements, Study Procedures, and Data Analysis

#### Demographic Information

Standardised face-to-face interviews with a researcher captured demographic and health information e.g., address, personal/household income and medication use. NZ Deprivation Index 2013 (NZDep2013) (Atkinson and Crampton, 2014) was derived from their geographical area of residence, which combines census data relating to home ownership, housing, qualifications, income, employment, access to transport, communications, and family structure. This was used as a measure of socioeconomic status, where a NZDep2013 score of one represents the areas “least deprived” and 10 “most deprived.”

#### Physical Activity

To objectively measure PA and sleep, participants wore a triaxial w-GT3X accelerometer (Actigraph, Pensacola, FL, United States) (Actigraph, 2013) on their non-dominant hip and an Acti-Watch (Micro Motionlogger^®^) on their non-dominant wrist, which are the standard placements for measuring movement (Ward et al., 2005) and sleep (Ancoli-Israel et al., 2003), respectively. Both devices were set to record an epoch length of 1 min. Participants were instructed to wear both devices continuously (24-h protocol) for the following eight days, except while bathing or participating in water activities such as swimming, thus not contributing toward the overall activity levels measured by the Actigraph. During the monitor wear period, participants completed a daily sleep and PA diary recording: (1) sleep onset; and end times for any sleep, including naps of ≥ 10 min duration; (2) any intentional PA (exercise) they engaged in, describing the type (e.g., running, pump class at gym, and walking); start time, duration and perceived intensity of the exercise session. Participants were asked to press the Acti-Watch “event” button when they went to bed and turned off their light to sleep, and when they woke up. This was to mark sleep onset and wake time in the memory of the Acti-Watch.

The w-GT3X accelerometer data were processed using Acti-Life^®^ software (version 6.13.3, Actigraph). Data from the first and last days (days of partial wear) of monitoring were excluded from analysis due to incomplete data. Data was focused on PA, using a midnight-midnight 24-h data format. All accelerometer epochs that occurred during sleep periods were identified using the sleep diaries and autographically confirmed using Acti-Watch data sleep times, recorded, and subsequently removed, leaving only epochs that occurred during waking hours for analysis. To autographically confirm sleep diaries a trained researcher visually inspected participant’s individual Acti-Watch download graph (using WatchWare software version 1.94.0.0) concurrently with the sleep diary. If the sleep diary stated the participant was sleeping and either: (1) the participant had pressed the sleep onset/wake button; or (2) the Acti-Watch graph visually indicated the participant was sleeping, this was confirmed as a correct sleep period. If no time was written in the sleep diary, the Acti-Watch time was used.

Non-wear time for the w-GT3X was defined as ≥ 60 consecutive minutes of zero epoch counts, with allowance for 2 min of counts between zero and 100. Participant’s data were considered valid if they wore the accelerometer for ≥ 12 h/day (Matthews et al., 2012), on ≥ 4 days, including one weekend day (Tudor-Locke et al., 2012). All invalid data were removed; the remaining epochs were categorised into time (min⋅day^–1^) spent in the following levels of PA (counts/min, cpm), using widely-used and validated cut-points (Troiano et al., 2008); sedentary behaviour (0–99), and light (100–2019), moderate (2,020–5,998) and vigorous intensity PA (≥5,999) and MVPA, a composite measure of moderate and vigorous PA (≥2,020). The average daily vector magnitude counts per minute (cpm) was calculated as an indicator of total PA volume (i.e., total daily movement).

#### Anthropometry

Height was measured to the nearest millimetre with a calibrated Harpenden stadiometer. Fasted body weight was measured on calibrated electronic scales (Sauter platform scale E1200, GmbH, Germany) to the nearest 0.01 kg. Hip and waist circumference were measured with a flexible steel tape (Lufkin W600PM) to the nearest 0.1 cm. For height, body weight, waist and hip circumference, two measurements were taken; if the second measurement was not within 1% of the first, a third measurement was taken. The mean value was recorded if two measurements were taken. If three measurements were taken the median value was recorded. Measured weight and height were used to calculate BMI (kg⋅m^–2^). Body composition including total body, android, gynoid and visceral fat was assessed using dual-energy X-ray absorptiometry (DXA) (Hologic QDR Discovery A, Hologic Inc. with APEX V. 3.2 software) by accredited researchers (Australian and NZ Bone Mineral Society clinical densitometry accreditation) (Kindleysides et al., 2019). Following a 10-min resting period, blood pressure and heart rate were measured in the supine position with a digital blood pressure monitor (Omron HEM-907, Omron Healthcare Inc.). Three consecutive measurements were taken at 1-min intervals; the mean of the second and third measurements was used for analysis (Egan et al., 2010).

#### Metabolic Health Risk Factors

Between the hours of 7:30 am and 9:00 am, trained phlebotomists drew 30 ml of blood from fasted participants (overnight, 10–15 h), to obtain serum and plasma for analysis of metabolic markers and endocrine regulators. Ethylenediaminetetraacetic acid (EDTA) vacutainers (Becton Dickinson) were used for whole blood and stored immediately at −80°C before the remainder of the sample was placed on wet ice until centrifugation. The serum vacutainer was left to stand for between 30 and 60 min at room temperature (18°C) to clot. For endocrine regulators, an additional plasma sample (Becton Dickinson vacutainer P800 EDTA, aprotinin, and dipeptidyl peptidase IV) was collected. All EDTA vacutainer tubes were centrifuged at 3500 rpm for 15 min at 4°C within 1 h of sample collection. Aliquots of plasma and serum were transferred into pre-labelled 1.5 mL microcentrifuge tubes (Eppendorf^®^ safe-lock PCR clean tubes, Hamburg, Germany) and cryovials (Cryo.S Greiner Bio-One, GmbH) and stored immediately at −80°C (Kindleysides et al., 2019).

Plasma levels of leptin were analysed (Plant & Food Research Mt Albert, Sandringham, NZ, United States), using the MILLIPLEX MAP Human Metabolic Hormone Magnetic Bead Panel 96-Well Metabolism Multiplex Assay (Millipore, United States, Cat # HMHEMAG-34K). Leptin samples were assayed in duplicate and plates were read using the Bioplex 100 Analyzer System (Bio-Rad). The leptin range was 0.37–66.0 ng/mL [inter-assay CV: 4.1%, intra-assay CV: 3.5%] and measurements had acceptable percentage of values above the limit of detection (98.4%).

Serum levels of insulin were measured using the electrochemiluminescence immunoassay (ECLIA) method (Roche Diagnostics, Mannheim, Germany) using the Cobas e411 analyser (Hitachi High Technologies Corporation, Tokyo, Japan). The inter-assay %CVs for insulin was 0.5%.

Total cholesterol and non-esterified fatty acids (NEFA) and triglycerides (Trig), HDL, low density lipoprotein (LDL), C-Reactive Protein (CRP), glucose and insulin, were measured using a Cobas e411 automatic electronic analyser (Roche, New Zealand), with kits supplied by Roche Diagnostics (Mannheim, Germany). Inter-assay coefficients of variation for total cholesterol, NEFA, Trig, HDL, LDL, CRP, glucose and insulin were 2.0, 3.4, 0.8, 6.1, 0.9, 3.3, 0.7, and 0.5%, respectively. EDTA whole blood was used to measure Glycated haemoglobin (HbA1c) levels by turbidimetric inhibition immunoassay (Roche Diagnostic, Mannheim, Germany) on a Hitachi c311 autoanalyser (Hitachi High Technologies Corporation, Tokyo, Japan). The inter-assay %CV for HbA1c was 1.0%. Insulin resistance was calculated using HOMA of insulin resistance (HOMA-IR) {HOMA-IR = [fasting plasma insulin (pmol/ml) × fasting blood glucose (mmol/L)]/22.5} (Wallace et al., 2004).

#### Statistical Analysis

All statistical analyses were performed using IBM SPSS software for Windows version 24.0 (SPSS Inc., Chicago, IL, United States). Normality of data was tested using histograms and Kolmogorov–Smirnov tests. Fasting insulin and CRP were logarithmically transformed (*ln*) to ensure a normal distribution. For descriptive statistical analysis, participants were categorised into two groups based on ethnicity (Pacific or NZE) and two further subgroups based on predetermined BF% criteria (low < 35 or high ≥ 35 BF%). Means and standard deviations (SD) were used to summarise all continuous data, except variables that were logarithmically transformed, which were presented as geometric means and geometric standard deviations (GSD). Frequencies (%) were reported for categorical variables. Differences between ethnic and BF% groups were measured with one-way ANOVA, with Bonferroni *post hoc* correction. Due to NZE women being significantly older (*p* < 0.05) and less deprived (*p* < 0.05) than Pacific women and considering age and deprivation are negatively associated with metabolic health (Ministry of Health New Zealand, 2020), all further analysis was carried out controlling for age and deprivation index (NZDep2013). Further adjustment for wear time did not materially affect the study outcomes (data not shown).

Separate multiple linear regression models were used to assess the association of MVPA and total PA with body composition and metabolic health markers, with adjustment for age and NZDep2013 as potential confounders. For assessing the independent association of MVPA and total PA with metabolic health markers, further adjustment for BF% group was conducted. Regression coefficients (β) obtained from log-transformed data represent relative differences and were expressed as a ratio (by using e^β^). For total daily PA we expressed the difference in body composition and metabolic health markers per 100 units increase of daily PA; for MVPA we expressed the difference per 10 units increase. Analyses were conducted separately for NZE and Pacific participants, as well as for both groups combined. For combined (NZE and Pacific) analyses, adjustments for ethnicity were made.

## Results

### Participant Characteristics

**FIGURE 1:**
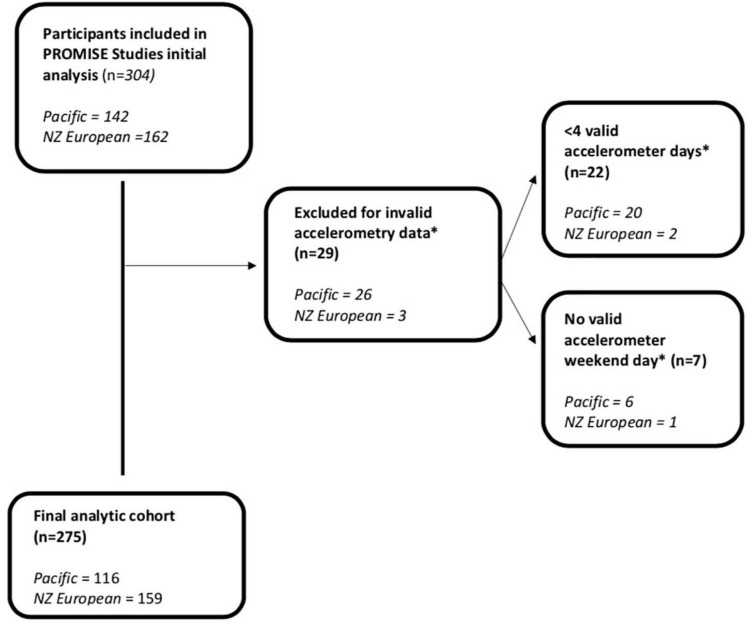
Flowchart of participants included in analysis.

Analyses were conducted on 275 women (Figure 1). PA, anthropometric and adiposity characteristics of participants are presented in Table 1. Pacific women were younger (*p* < 0.05) and had higher NZDep2013 scores than NZE women (*p* < 0.05).

**TABLE 1:** Characteristics and difference in anthropometry, accelerometery, dietary, socioeconomic, and endocrine by ethnicity and body fat% group.

**Characteristic**	**Pacific**		**NZ European**	
	**<35% body fat**	**≥35% body fat**	**<35% body fat**	**≥35% body fat**
		
	**Mean** (**SD**)	**Mean** (**SD**)	**Mean** (**SD**)	**Mean** (**SD**)
*N* (%)	61 (22)	55 (20)	85 (31)	74 (27)
BMI (kg⋅m^–2^)	25.9 (3.9)^†^^∧^	35.6 (6.1)	22.5 (2.1)^†^	33.7 (3.8)
Age (years)	25 (7)^∧^	26 (6)^∧^	30 (7)^†^	33 (7)
***Socioeconomic***				
NZDep2013	7 (3)^∧^	8 (2)^∧^	4 (2)	5 (2)
***Anthropometry***				
Weight (kg)	73.70 (11.76)^†^^∧^	99.65 (18.30)	60.06 (7.62)^†^	94.40 (13.30)
Height (cm)	168.78 (6.57)	167.33 (7.32)	167.19 (5.61)	167.19 (7.05)
Waist circumference (cm)	79.64 (8.01)^†^^∧^	98.50 (13.30)	73.22 (5.75)^†^	97.98 (9.92)
Hip circumference (cm)	105.15 (7.06)^†^^∧^	121.37 (10.84)	98.02 (6.75)^†^	120.40 (8.75)
Systolic blood pressure	111.4 (9.3)^†^	118.7 (11.4)	112.7 (10.6)^†^	120.3 (13.7)
Heart rate (beats/minute)	69.4 (11.2)	70.5 (11.1)	67.1 (11.4)	74.3 (12.0)
Total body fat (kg)	22.44 (4.99)^†^^∧^	39.59 (9.80)	17.83 (4.29)^†^	39.04 (7.68)
Total body fat (%)	30.10 (3.07) ^†^^∧^	39.48 (3.46)	27.84 (4.46)^†^	41.16 (3.78)
Total lean mass (kg)	51.62 (7.42)^†^^∧^	59.80 (9.18)^∧^	45.75 (5.26)^†^	55.31 (7.15)
Android fat mass (kg)	4.70 (1.02) ^†^^∧^	7.30 (1.89)	4.10 (0.74)^†^	7.22 (1.35)
Android fat mass (%)	29.33 (5.09)^†^^∧^	41.52 (4.82)	24.78 (5.18)^†^	41.50 (4.90)
Gynoid fat mass (kg)	12.66 (2.09)^†^^∧^	16.88 (2.93)	11.02 (1.63)^†^	16.21 (2.55)
Gynoid fat mass (%)	35.05 (3.39)^†^	41.02 (3.49)	33.34 (4.20)^†^	42.50 (4.19)
Visceral fat (%)	27.06 (5.52)^†^^∧^	39.56 (5.03)	22.15 (5.91)^†^	39.65 (5.15)
***Accelerometery***				
Valid days (*n*)	6 (1)	6 (1)	6 (0)	6 (0)
Participants with only four valid days (*n*)	1	2	2	1
Waking wear time (h day^–1^)	15.6 (1.2)	15.6 (1.3)	15.2 (1.0)	15.2 (1.1)
Minimum waking wear time (h day^–1^)	13.0	12.2	13.2	12.9
Light PA (min⋅day^–1^)	314.1 (76.9)	342.2 (97.7)	328.3 (79.4)	320.9 (85.7)
Sedentary behaviour (min⋅day^–1^)	596.3 (85.0)^∧^	574.5 (98.9)	545.8 (75.3)	560.2 (91.5)
MVPA (min⋅day^–1^)	26.3 (15.6)^∧^	19.1 (15.2)^∧^	39.1 (18.4)^†^	30.52 (19.1)
Moderate PA (min⋅day^–1^)	21.5 (13.6)^∧^	18.2 (14.1)^∧^	34.6 (15.3)	29.3 (18.8)
Vigorous PA (min⋅day^–1^)	1.9 (5.2)^∧^	0.8 (1.7)	4.9 (6.1)^†^	1.2 (3.0)
Sleep duration (min⋅day^–1^)	456.0 (64.5)	431.2 (62.7)^∧^	477.0 (49.4)	461.3 (46.5)
Total daily PA (cpm)	598 (168)^∧^	607 (185)	731 (179)	674 (210)
***Carbohydrate metabolism***				
Glucose (mmol/L)	5.31 (0.48)^∧^	5.47 (0.51)	5.12 (0.31)^†^	5.52 (0.46)
HbA1c (mmol/mol)	32.21 (1.99)^†^^∧^	34.10 (2.97)^∧^	30.44 (2.19)^†^	31.62 (2.81)
HOMA-IR	21.6 (20.1)^†^^∧^	39.4 (38.0)^∧^	10.2 (4.5)^†^	22.4 (13.3)
***Lipid profile***				
Chol (mmol/L)	4.60 (0.72)	4.74 (0.78)^∧^	4.92 (0.86)^†^	5.40 (1.18)
HDL (mmol/L)	1.58 (0.32)^†^^∧^	1.40 (0.32)	1.84 (0.37)^†^	1.50 (0.32)
LDL (mmol/L)	2.83 (0.66)	2.99 (0.73)^∧^	2.89 (0.83)^†^	3.53 (1.16)
NEFA (mmol/L)	0.66 (0.32)	0.81 (0.35)	0.67 (0.32)	0.77 (0.38)
Trig (mmol/L)	0.89 (0.34)^†^	1.25 (0.64)	0.78 (0.28)^†^	1.20 (0.51)
***Endocrine regulators***				
Leptin (pg/ml)	9471 (6605)^†^	23284 (10922)	6037 (4166)^†^	26852 (13327)
Insulin (pmol/ml)^#^	73.04 (1.76) ^†^^∧^	122.82 (1.94)^∧^	40.74 (1.51)^†^	79.84 (1.62)
***Inflammation marker***				
CRP (mg/L)^#^	0.49 (2.56)	1.19 (2.72)^∧^	0.74 (2.81)	2.62 (2.52)

*Values are presented as mean ± standard deviation unless otherwise specified; ^#^geometric mean ± geometric standard deviation); ^†^*p* < 0.05 for test comparing body fat groups within ethnic group; ^∧^*p* < 0.05 for test comparing between ethnic groups within body fat group. BMI, Body mass index; NZDep2013, NZDep2013 index of socioeconomic deprivation; PA, physical activity; Light PA, light intensity physical activity; MVPA, moderate to vigorous physical activity; HbA1c, fasting blood glycated haemoglobin; HOMA-IR, Homeostatic Model Assessment of Insulin Resistance; CRP, fasting C Reactive Protein; Chol, fasting blood total cholesterol; HDL, fasting High-density lipoprotein; LDL, fasting Low-density lipoprotein; NEFA, fasting non-esterified fatty acids; Trig, fasting triglycerides; Pacific *n* = 2 with missing deprivation data, NZE *n* = 1 with missing deprivation data.*

### Physical Activity

A weak positive Pearson’s correlation was found between MVPA and LIPA; *r* = 0.178 (*p* < 0.01). Participants achieved a mean of 6 days of valid accelerometer recordings. All four groups’ mean waking wear time was > 15 h per day and there was no significant difference between groups: Pacific high- [15.6 (1.3) h day^–1^] and low-BF% [15.6 (1.2) h day^–1^] and NZE high- [15.2 (1.1) h day^–1^] and low-BF% [15.2 (1.0) h day^–1^; Table 1].

On average participants spent the majority of the waking day in sedentary behaviour (546–596 min⋅day^–1^) (Table 1). When comparing means, light intensity PA (LIPA) was the main intensity of PA in all groups and there was no difference in time spent in LIPA between ethnic or BF% groups. All groups spent less than 3% of the 24-h day in MVPA, and differences were found between BF% (*p* ≤ 0.05) and ethnic (*p* ≤ 0.05) groups: Pacific high- (19 min⋅day^–1^) and low-BF% (26 min⋅day^–1^) and NZE high- (31 min⋅day^–1^) and low-BF% (39 min⋅day^–1^; Table 1).

Pacific women in the low-BF% group engaged in significantly less total PA when compared to NZE women in the low-BF% group (*p* ≤ 0.05), however there was no ethnic difference in total PA between high-BF% groups (Table 1).

### Body Composition

Both Pacific and NZE women’s weight, waist and hip circumferences and BMI were significantly higher in the high-BF% group when compared to the low-BF% groups (*p* < 0.05; Table 1). Pacific women weighed more (*p* < 0.05) and had a larger hip (*p* < 0.05) and waist (*p* < 0.05) circumferences when compared to NZE women. There was no ethnic difference in total or regional BF% in the high BF% group; however, when comparing the low-BF% groups, NZE women had lower total (*p* < 0.05), android (*p* < 0.05), gynoid (*p* < 0.05), visceral (*p* < 0.05), and trunk (*p* < 0.05) fat percentages.

### Biomarkers of Metabolic Health

Pacific women and women with high-BF% had significantly higher HbA1c and fasting insulin concentrations when compared to NZE women and women with low-BF%. Mean plasma glucose was higher among Pacific women with a low-BF% than for NZE with a low-BF% (*p* < 0.05). Glucose was also significantly higher for NZE with a high-BF% when compared to their low-BF% counterparts; Pacific high-BF%, 5.47 mmol/L; and low-BF% 5.31 mmol/L; NZE high-BF%; 5.52 mmol/L; and low-BF%, 5.12 mmol/L (Table 1). Among women with a high-BF% NZE women had increased CRP when compared to Pacific women (*p* < 0.05). Both NZE and Pacific women’s circulating concentrations of triglycerides were significantly higher in the high-BF% group when compared to the low-BF% group and there was no significant ethnic difference. Among high-BF% women, Pacific had significantly lower LDL concentrations compared to NZE. NZE low-BF% women also had significantly lower LDL concentrations compared to NZE high-BF% women. Both Pacific and NZE women’s leptin concentrations were significantly higher in the high-BF% group when compared to the low-BF% groups (Table 1), which parallels the BF% measurements described above.

### Multiple Linear Regression Analysis

Multiple linear regression analysis showed a significant albeit small inverse association between total PA, and NZE women’s total body, trunk, visceral, android and gynoid fat% (Table 2). Every 100 cpm increase in total daily PA was associated with 0.7% lower gynoid fat, 0.8% lower total body, trunk and android and 0.9% lower visceral body fat (Table 2). However, we observed no significant association between total PA and any measure of body composition in Pacific women. When investigating time spent in MVPA, we observed a significant association with lower total, trunk and gynoid BF% in both NZE and Pacific women, with the associated lower BF% being of the same magnitude in both ethnic groups (Table 2). Every additional 10 min spent in MVPA was associated with 0.9% lower total and trunk fat and 0.7% lower gynoid fat. In addition, every 10-min increase in MVPA was inversely associated with android (0.9%) and visceral (1.0%) BF% in NZE women only (*p* ≤ 0.05).

**TABLE 2:** Association between increasing total physical activity (cpm) and MVPA (min/day) and body composition.

**Body region**	**Pacific**		**NZ European**		**All participants ≠**	
	***n* = 114**		***n* = 158**		***n* = 272**	
	**Total daily PA, difference (95% CI) per 100 units increase**	**MVPA, Difference (95% CI) per 10 units increase**	**Total daily PA, difference (95% CI) per 100 units increase**	**MVPA, difference (95% CI) per 10 units increase**	**Total daily PA, difference (95% CI) per 100 units increase**	**MVPA, difference (95% CI) per 10 units increase**
Trunk fat%	−0.5 (−1.2, 0.3)	−0.95 (−1.80, −0.10)*	−0.8 (−1.5, −0.1)*	−0.91 (−1.63, −0.19)*	−0.7 (−1.2, −0.2)*	−0.95 (−1.49, −0.40)**
Android fat%	−0.5 (−0.4, 0.3)	−0.90 (−1.84, 0.05)	−0.8 (−1.6, 0.0)*	−0.94 (−1.72, −0.16)*	−0.7 (−1.2, −0.1)*	−0.95 (−1.54, −0.36)**
Gynoid fat%	−0.4 (−0.9, 0.1)	−0.65 (−1.20, −0.10)*	−0.7 (−1.2, −0.2)**	−0.65 (−1.15, −0.16)**	−0.6 (−0.9, −0.2)**	−0.67 (−1.04, −0.31)***
Visceral fat%	−0.5 (−0.4, 0.3)	−0.91 (−1.90, 0.07)	−0.9 (−1.7, 0.0)*	−1.00 (−1.83, −0.17)*	−0.7 (−1.3, −0.1)*	−0.99 (−1.62, −0.37)**
Total body fat%	−0.4 (−0.0, 0.2)	−0.89 (−1.56, −0.21)*	−0.8 (−1.4, −0.2)**	−0.85 (−1.47, −0.23)**	−0.6 (−1.1, 0.2)**	−0.88 (−1.33, −0.42)***
Waist circumference (cm)	−0.5 (−1.9, 1.0)	−1.37 (−3.05, 0.31)	−0.6 (−1.8, 0.5)	−1.39 (−2.52, −0.27)*	−0.7 (−1.6, 0.2)	−1.58 (−2.46, −0.70)***
BMI (kg m^–2^)	0.1 (−0.7, 0.8)	−0.46 (−1.30, 0.37)	−0.3 (−0.8, 0.2)	−0.53 (−1.03, −0.4)*	−0.3 (−0.7, 0.1)	−0.64 (−1.05, −0.23)**

Total *n* = 272: Pacific *n* = 114 (*n* = 2 with missing NZDep2013 data), NZE *n* = 158 (*n* = 1 with missing NZDep2013 data) Abbreviations: NZ, New Zealand; PA, physical activity; MVPA, moderate to vigorous physical activity. All models adjusted for age and NZDep2013; ≠ model further adjusted for ethnicity; *p < 0.05, **p < 0.01, ***p < 0.001. Regression coefficients represent the change in the outcome per 100 cpm change in total daily PA and 10 min change in MVPA.

Among Pacific women, significant inverse associations were found between total PA and fasting plasma insulin. Every 100 cpm increase in total PA was associated with a 6% lower fasting plasma insulin; this association was not significant in NZE women (Table 3). For both Pacific and NZE women, every 10-min increase in MVPA was associated with 8% lower fasting plasma insulin (*p* ≤ 0.05) (Table 3).

**TABLE 3:** Association between total daily PA (cpm) and MVPA (min/day) and metabolic health markers.

**Biomarker**	**Pacific**		**NZ European**		**All participants^≠^**	
**Endocrine**	**Total daily PA, difference (95% CI) per 100 units increase**	**MVPA, difference (95% CI) per 10 units increase**	**Total daily PA, difference (95% CI) per 100 units increase**	**MVPA, difference (95% CI) per 10 units increase**	**Total daily PA, difference (95% CI) per 100 units increase**	**MVPA, difference (95% CI) per 10 units increase**
HbA1c (mmol mol)	−0.1 (−0.3, 0.2)	0.1 (−0.2, 0.4)	0.2 (0.0, 0.4)*	−0.1 (−0.3, 0.1)	0.1 (0.0, 0.1)	0.0 (−0.2, 0.2)
Glucose (mmol L)	0.0 (−0.1, 0.0)	−0.01 (−0.07, 0.05)	0.0 (0.0, 0.0)	−0.03 (−0.06, 0.00)	0.0 (0.0, 0.0)	−0.02 (−0.05, 0.01)
HOMA-IR	−2.8 (−5.9, 0.4)	−2.6 (−6.3, 1.0)	0.2 (−0.6, 0.9)	−0.9 (−1.7, −0.1)*	−0.9 (−2.2, 0.4)	−1.5 (−2.9, −0.03)*
Leptin (pg ml)	−729 (−1711, 251)	−989 (−2090, 112)	−26 (−826, 774)	31 (−794, 857)	−304 (−917, 310)	−267 (−920, 387)
Chol (mmol L)	0.0 (−0.1, 0.1)	0.05 (−0.03, 0.14)	0.0 (−0.1, 0.1)	0.03 (−0.03, 0.09)	0.0 (0.0, 0.1)	0.01 (−0.05, 0.07)
NEFA (mmol L)	0.0 (0.0, 0.1)	−0.01 (−0.08, 0.05)	0.0 (0.0, 0.0)	−0.03 (−0.06, 0.00)	0.0 (0.0, 0.0)	0.02 (0.00, 0.05)
Trig (mmol L)	0.0 (−0.1, 0.0)	−0.01 (−0.08, 0.05)	0.0 (0.0, 0.0)	−0.03 (−0.06, 0.00)	0.0 (−0.1, 0.0)	−0.03 (−0.06, 0.01)
HDL (mmol L)	0.0 (0.0, 0.1)	0.03 (0.00, 0.07)	0.0 (0.0, 0.0)	0.04 (0.02, 0.06)***	0.0 (0.0, 0.0)	0.03 (0.01, 0.06)**
LDL (mmol L)	0.0 (−0.1, 0.1)	0.03 (−0.06, 0.11)	0.0 (0.1, 0.1)	0.01 (−0.05, 0.06)	0.0 (0.0, 0.1)	0.00 (−0.06, 0.06)
***Anthropometry***						
Weight (Kg)	0.4 (−1.2, 2.0)	0.49 (−1.49, 2.38)	0.3 (−0.6, 1.2)	−0.12 (−1.03, 0.78)	0.3 (−0.5, 1.1)	0.12 (−0.77, 1.01)
Waist circumference (cm)	−0.5 (−1.6, 0.6)	−0.50 (−1.79, 0.79)	0.4 (−0.2, 1.1)	−0.14 (−0.81, 0.52)	0.1 (−0.5, 0.6)	−0.22 (−0.85, 0.41)
Hip circumference (cm)	0.1 (−0.9, 1.0)	0.22 (−0.90, 1.33)	0.1 (−0.6, 0.7)	−0.04 (−0.70, 0.62)	0.0 (−0.5, 0.6)	0.09 (−0.49, 0.66)
Systolic blood pressure (mmHg)	1.1 (0.1, 2.2)*	0.45 (−0.79, 1.70)	0.1 (−1.0, 1.1)	−0.30 (−1.34, 0.74)	0.4 (−0.3, 1.1)	0.00 (−0.79, 0.79)
Diastolic blood pressure (mmHg)	0.5 (−0.3, 1.4)	−0.17 (−1.20, 0.86)	−0.5 (−1.2, 0.3)	−0.045 (−0.122, 0.031)	−0.2 (−0.7, 0.4)	−0.29 (−0.90, 0.33)
Heart rate (bpm)	−1.1 (−2.3, −0.2)	−1.83 (−3.19, −0.48)**	−1.4 (−2.4, 0.4)**	−1.21 (−2.26, −0.17)*	−1.2 (−2.0, −0.4)**	−0.14 (−2.26, −0.63)**

	**Total daily PA, ratio (95% CI) per 100 units increase**	**MVPA, ratio (95% CI) per 10 units increase**	**Total daily PA, ratio (95% CI) per 100 units increase**	**MVPA, ratio (95% CI) per 10 units increase**	**Total daily PA, ratio (95% CI) per 100 units increase**	**MVPA, ratio (95% CI) per 10 units increase**

Insulin^†^ (pmol/ml)	0.94 (0.88, 1.00)	0.92 (0.85, 0.99)*	1.00 (1.00, 1.00)	0.92 (0.90, 0.96)***	1.00 (0.91, 1.00)*	0.95 (0.91, 0.98)**
CRP^†^ (mg L)	0.91 (0.82, 1.00)	0.96 (0.85, 1.10)	0.91 (0.82, 1.00)*	0.90 (0.83, 0.98)*	0.91 (0.82, 1.00)**	0.92 (0.87, 0.99)*

Total *n* = 272: Pacific *n* = 114 (*n* = 2 with missing deprivation data), NZE *n* = 158 (*n* = 1 with missing deprivation data). NZE, New Zealand European; PA, physical activity; MVPA, moderate to vigorous physical activity; HbA1c, fasting blood glycated haemoglobin; HOMA-IR, Homeostatic Model Assessment of Insulin Resistance; CRP, fasting C Reactive Protein; Chol, fasting blood total cholesterol; HDL, fasting High-density lipoprotein; LDL, fasting Low-density lipoprotein; NEFA, fasting non-esterified fatty acids; Trig, fasting triglycerides. All models adjusted for body fat% group, age and NZDep2013; ^≠^further adjusted for ethnicity; *p < 0.05, **p < 0.01, ***p < 0.001; ^†^data has been log transformed (*ln*). Regression coefficients represent the change in the outcome per 100 cpm change in total daily PA and 10 min change in MVPA.

Among NZE women every 100 cpm increase in total PA and 10-min increase in MVPA was significantly associated with 9% and 10% lower (*p* ≤ 0.05) fasting CRP concentration, respectively (Table 3); no significant association was observed in Pacific women.

A significant inverse association was also found between increasing total PA and heart rate for NZE women: every 100 cpm increase in daily PA was associated with a lower resting heart rate of 1.4 beats per minute (*p* < 0.05). Further, every additional 10 min spent in MVPA was significantly associated with a lower heart rate in Pacific and NZE women (1.8 and 1.2 beats/min, respectively) and significantly lower HOMA-IR (0.36) among NZE women. Small significant positive associations were also observed with increasing total PA and Pacific women’s systolic blood pressure and NZE women’s HbA1c concentrations (*p* < 0.05; Table 3).

## Discussion

Results from this study indicate, that in a healthy population, increasing PA, in particular MVPA, is associated with improved blood glucose metabolism, lower heart rate and BF% for Pacific and NZE women, despite different metabolic disease risk, body fat profiles and deprivation level.

### Physical Activity and Body Composition

In this study, increased MVPA was associated with lower total body, trunk and gynoid fat% in both Pacific and NZE women. A large amount of cross-sectional, longitudinal and intervention research has been carried out investigating the associations between MVPA and body composition. However, systematic reviews and meta-analysis collating this evidence report equivocal results (Fogelholm and Kukkonen-Harjula, 2000; Franz et al., 2007; Wilks et al., 2011; Hebden et al., 2012; Baillot et al., 2014; Conn et al., 2014), which may be due to differences in the experimental design and methodology making studies difficult to compare: Baillot et al. (2014) only investigated individuals with obesity, some only considered weight gain as an outcome (Fogelholm and Kukkonen-Harjula, 2000; Hebden et al., 2012), others assessed body composition through BMI (Wagner et al., 2001; Britton et al., 2012; Ekelund et al., 2017; Gibbs et al., 2017) and waist circumference (Wagner et al., 2001; Gibbs et al., 2017), whilst some investigated more detailed measures of body composition such as BF% (Wilks et al., 2011; Baillot et al., 2014; O’Brien, 2018). In a similar population of NZ European and Pacific women, who engaged in similar amounts of MVPA to the current study (35 and 22 min/day respectively), O’Brien (2018), found no association between increasing MVPA and any BF% region they measured using DXA (gynoid, android, whole BF%) in either Pacific or NZ European women (O’Brien, 2018). In this study, higher total PA was associated with significantly lower total, trunk, android, gynoid and visceral BF% amongst NZE women. Although these same trends were observed amongst Pacific women, results were not significant. Research investigating associations between total PA and BF% is scarce, thus it is difficult to compare to previous research. None-the-less, Wolff-Hughes (2015) found similar results to our NZE women, reporting significant inverse associations with total PA and triceps and subscapular skinfold measurements.

A notable finding was the significant inverse association between increasing total PA and MVPA and visceral and android fat% amongst NZE, but not Pacific women. Pacific and NZE women had similar total body fat mass; however, low-BF% Pacific women had significantly higher visceral adiposity when compared to their NZE counterparts. This finding is important, as higher visceral adiposity has long been associated with increased risk of non-communicable diseases and inflammation (Fontana et al., 2007; Fox et al., 2007; Stępień et al., 2014; Kang et al., 2015; Zhang et al., 2015), and thus may contribute toward Pacific women’s increased susceptibility to metabolic diseases (Healy et al., 2008; Henson et al., 2013; Swindell et al., 2018). Pacific women may therefore require higher levels of total PA and/or MVPA to experience a similar decrease in total and regional BF% as observed in NZE women.

### Physical Activity and Biomarkers of Metabolic Health

High-BF% was associated with significantly higher CRP concentrations (Table 1). This is suggestive of the influence of adipose tissue on the pro-inflammatory state (Fontana et al., 2007). It is interesting to note that Pacific women had significantly lower CRP levels compared to NZE women; this is despite having a similar total BF%. Also, Pacific women in this study had fasting plasma insulin concentrations almost two-fold higher than their NZE counterparts. Our results are consistent with other research comparing insulin concentrations and CRP levels of NZE and Pacific women (McAuley et al., 2002; Whitford, 2017; O’Brien, 2018; Murphy et al., 2019). It is important to note that participants in this study were “healthy” and had not been diagnosed with a chronic disease. Mean CRP for both ethnic groups were < 5.0 mg/L, suggesting no presence of clinical inflammation. Further, the increased insulin concentrations observed in Pacific women of the present study did not appear to have elicited a chronic pathophysiological response [i.e., all participants had normal HbA1c level below 41 mmol/mol (Labtests, 2021)]. Further, it is well documented that sustained increased levels of insulin may indicate a reduced degree of insulin sensitivity; therefore, the observed profile may be indicative of the preliminary stages of insulin hypersecretion and early signs of reduced insulin sensitivity among these Pacific women. Previous research has shown that, when compared to NZE, South Pacific people (NZ Māori) had lower insulin sensitivity (measured using euglycemic insulin clamp) at an equivalent level of BMI (McAuley et al., 2002). However, this population consisted of NZ Māori (despite being described as South Pacific people), and metabolic risk (or profiles) is different between NZ Māori and other Polynesian groups, such as participants in our study (Merriman and Wilcox, 2018). Whilst the mechanisms underlying this observation cannot be determined from this study, ethnic differences in the low BF% group’s fasting insulin concentrations may in part be explained by their higher visceral adiposity; this, however, does not explain the ethnic differences in high BF% groups fasting insulin concentrations. However, two important findings of this study may also help explain these ethnic differences. Firstly, Pacific women in the high BF% group engaged in significantly less MVPA when compared to the NZE group, and secondly, MVPA was significantly associated with lower insulin in both ethnic groups. This emphasises the importance of encouraging all women to engage in PA, including MVPA.

Although the positive associations found between increasing total PA and Pacific women’s systolic blood pressure and NZE women’s HbA1c concentrations should be investigated further, they are unlikely to be clinically relevant and may be a statistical anomaly. For example, if Pacific women in this study were to increase their total PA by as much as 50%, they would only see a 3.3 mmHg increase in their systolic blood pressure (mean total daily PA; 598 and 607 cpm in the low- and high-BF% groups respectively). Further, for NZE participant’s HbA1c to increase by as little as 1.0 mmol/mol they would need to increase their total daily PA by 500 cpm, which equates to an increase of at least 75%.

Within our population, there was an inverse association between total PA and key markers of metabolic health (insulin, CRP, and heart rate). Other research in European populations has also reported increased total PA to be significantly positively associated with metabolic health including fasting insulin, HOMA_IR, and CRP (Ekelund et al., 2007; Swindell et al., 2018), thus it is promising to also see metabolic benefits of total PA in other ethnic groups (i.e., Pacific) with higher metabolic disease risk. It is particularly interesting to observe a significant inverse association between total PA and fasting insulin concentrations among Pacific, but not NZE women. This association may be explained by fasting plasma insulin concentrations among Pacific women being two-fold higher than those of the NZE women, hence the capacity for change was greater among Pacific women. This is an important finding considering Pacific women’s total PA was significantly lower than NZE women’s and it was not associated with body composition. Similarly, it was interesting to find no association between Pacific women’s CRP and total PA or MVPA, whereas significant negative associations among NZE women were observed. CRP is positively associated with visceral BF% (Sanip et al., 2013), which may explain the finding that increasing total PA and MVPA was inversely associated with visceral fat% amongst NZE women, but not in our Pacific population. Others report high levels of exercise and energy restriction leading to significant weight loss including reduction in visceral fat mass can improve cardiometabolic profile through inflammation-related biomarkers including CRP (Sarin et al., 2019).

Considering there is growing concern for the impact of sedentary behaviour on metabolic health, independent of PA, it is encouraging to see these associations between PA, adiposity and metabolic health in a sedentary population. Women in the current study were sedentary for a substantial portion of their waking day (>9 h/day). Comparable to our population, Canadian adults spend about 9–10 h/day in sedentary behaviour (Prince et al., 2020). However, other populations are considerably less sedentary (Matthews et al., 2008; Beale et al., 2020). Also using accelerometery data, Beale et al. (2020) investigated a similar population of NZ women to the current study, where participants spent an average of 7 h 42 min/day in SB (Beale et al., 2020). In comparison, data from the U.S. National Health and Nutrition Examination Survey (NHANES) indicate that approximately 55% of children’s and adult’s awake time (7.7 h d^–1^) is spent being sedentary (Matthews et al., 2008), which is over 1 h less than women in the current study.

Pacific women in this study spent less time in PA when compared to NZE women, which is consistent with previous research (O’Brien, 2018; O’Brien et al., 2019). O’Brien (2018) also investigated Pacific and NZE women’s objectively measured PA patterns and found participants engaged in similar amounts of MVPA to the current study (Pacific; 22 and NZE; 35 min/day) (O’Brien, 2018). The NZE women’s mean moderate PA levels fell within the recommended 150–300 min⋅week^–1^ (low-BF% 240 and high-BF% 205 min⋅week^–1^) whereas only the Pacific low BF% group met this guideline (low-BF% 150 and high-BF% 128 min⋅^–1^). This may help to explain the stronger inverse association between MVPA, total PA and adiposity and the majority of markers of metabolic health (HOMA_IR, CRP, HDL) observed in NZE women. However, it is important to note that this result may also be due to greater variance of PA measures (total daily PA and MVPA) observed in NZE women compared to Pacific women, thus resulting in more statistical power for the analyses involving NZE women.

Research has shown that a large portion of the association between socioeconomic position and obesity is mediated through health behaviours such as diet and PA (Pampel et al., 2010; Shaikh et al., 2015; Foster et al., 2018). The more deprived, the more vulnerable people are to the obesogenic environment, access to food and PA which is limited by cost, time and geographical locations (Exeter et al., 2017). Areas of high deprivation may have smaller tax income to fund recreational PA facilities, whilst simultaneously having higher crime rates, a barrier to engaging in PA in public spaces (Exeter et al., 2017).

Our results may suggest that PA is more effective at improving insulin and CRP in one ethnic group when compared to another but may depend on baseline insulin concentrations and CRP levels. Future studies could address pathways of insulin secretion, chronic systemic inflammation or the gut microbiome (Mailing et al., 2019), all of which have been implicated as pathways to obesity and increased metabolic disease risk.

### Strengths and Limitations

The objective measurement of PA is a strength to this study, as does the 24-h accelerometer wear protocol which allowed for objective measurement of sleep onset and wake times. Consequently, we could accurately remove all sleep data from the PA analysis without relying on algorithms to predict this data. However, we acknowledge wearing an accelerometer may in some individuals result in an increase of PA, particularly on the first day of monitoring (Baumann et al., 2018). To reduce the potential of overestimating average PA levels we have excluded data from the first day of monitoring; however, we cannot exclude the possibility that wearing an accelerometer may have also affected PA in subsequent days. Nonetheless, we would not expect this to be different between low and high BF% groups and Pacific and NZE women. Therefore, we do not believe that any increases in PA due to wearing an accelerometer would have resulted in significant bias.

The 24-h day is finite, therefore the time spent in all movement behaviours is co-dependent (sleep, sedentary behaviour, light intensity PA and MVPA); associations we observed between PA, body composition and metabolic markers of health may have reflected the highly sedentary nature of this population. Future studies should consider using statistically advanced analytical methods such as compositional analysis that account for this (Chastin et al., 2015).

There is no formal agreement on defining obesity in terms of BF% (Oliveros et al., 2014). However, a wide variety of BF% cut-off points have been used for women, varying between 30 to 37% (Oliveros et al., 2014). The chosen cut-point of for categorising high vs. low BF% (35%) was confirmed after carrying out analysis within our population comparing BF% with BMI. In particular, scatter plots, graphing BMI vs. BF% with a line of best fit suggested using 35% BF as our cut-point. Using this cut point only five participants with a BMI < 25 kg/m^2^ were in the > 35% BF group, suggesting potential misclassification in terms of BMI was minor. Further, cross-sectional designs are susceptible to reverse causality. As PA behaviour may change as a consequence of obesity, caution must be taken when interpreting these results. It is also possible that PA behaviours cluster with other unhealthy activities such as dietary or sleep patterns or increased sedentary behaviour.

## Conclusion

Objectively measured PA was associated with lower metabolic risk markers in both Pacific and NZE women. Fasting insulin concentrations differed between ethnic and BF% groups with an increased risk of hyperinsulinemia in Pacific women and women with obesity. Increases in total daily PA and MVPA were associated with lower fasting plasma insulin and CRP as well as heart rate, thus indicating a lower metabolic disease risk. These findings confirm the importance of PA but suggest the potential health benefit may differ between ethnic groups. Importantly, increased total PA appears to have a greater impact on Pacific women’s fasting insulin concentrations when compared to NZE. Considering Pacific women are a high metabolic disease risk population and 16% are diagnosed with T2D (Ministry of Health New Zealand, 2020), promotion of PA in this population should remain a priority.

## Data Availability Statement

The raw data supporting the conclusions of this article will be made available by the authors, without undue reservation.

## Ethics Statement

The studies involving human participants were reviewed and approved by Health and Disability Ethics Committee (Reference No. 16/STH/32). The patients/participants provided their written informed consent to participate in this study.

## Author Contributions

JS: conceptualization of the manuscript, data curation, and formal analysis. JS, RK, BB, JD, and MC: methodology. JS, BB, and RK: writing – original draft preparation. JS, JM-C, WO’B, JD, MC, RK, and BB: writing – review and editing. BB, RK, and JD: PROMISE study funding acquisition. All authors have read and agreed to the published version of the manuscript.

## Conflict of Interest

The authors declare that the research was conducted in the absence of any commercial or financial relationships that could be construed as a potential conflict of interest.
